# Cell–cell interactions as predictive and prognostic markers for drug responses in cancer

**DOI:** 10.1186/s13073-025-01518-5

**Published:** 2025-10-08

**Authors:** Xuehan Lu, Xiao Tan, Eun Ju Kim, Xinnan Jin, Meg L. Donovan, Jazmina L. Gonzalez Cruz, Zherui Xiong, Maria Reyes Becerra de los Reyes Becerra Perez, Jialei Gong, James Monkman, Divya Agrawal, Arutha Kulasinghe, U. Q. CellOmics Innovation Forum, Quan Nguyen, Zewen Kelvin Tuong

**Affiliations:** 1 Frazer Institute, Faculty of Health, Medicine and Behavourial Sciences, The University of Queensland, Brisbane, QLD 4102 Australia; 2 Institute for Molecular Biosciences, The University of Queensland, Brisbane, QLD 4067 Australia; 3 Division of Radiation Biomedical Research , Korea Institute of Radiological and Medical Sciences, Seoul, 01812 Republic of Korea; 4 Department of Radiological and Medico-Oncological Sciences, University of Science and Technology, Daejeon, 34113 Republic of Korea; 5 Queensland Spatial Biology Centre, Wesley Research Institute, The Wesley Hospital, Level 8 East Wing, Auchenflower, QLD 4066 Australia; 6 QIMR Berghofer Medical Research Institute, Brisbane, QLD 4006 Australia; 7 Australian Institute for Bioengineering and Nanotechnology, The University of Queensland, Brisbane, QLD 4067 Australia; 8 Department of Chemical Engineering, Indian Institute of Technology Delhi, Hauz Khas, New Delhi, 110016 India; 9 The University of Queensland, Brisbane, QLD 4072 Australia; 10 Ian Frazer Centre for Children’s Immunotherapy Research, Child Health Research Centre, Faculty of Health, Medicine and Behavourial Sciences, The University of Queensland, Brisbane, QLD 4101 Australia; 11 National Centre for Spatial Tissue and AI Research – NCSTAR, QIMR Berghofer Medical Research Institute, Brisbane, QLD 4006 Australia

**Keywords:** Cell–cell interactions, Cell–cell colocalization, Drug responses, Cancer, Tumor microenvironment, Spatial-omics, Single cell-omics, Precision treatment, Personalized therapy

## Abstract

The tumor microenvironment (TME) is composed of a diverse and dynamic spectrum of cell types, cellular activities, and cell–cell interactions (CCI). Understanding the complex CCI within the TME is critical for advancing cancer treatment strategies, including modulating or predicting drug responses. Recent advances in omics technologies, including spatial transcriptomics and proteomics, have allowed improved mapping of CCI within the TME. The integration of omics insights from different platforms may facilitate the identification of novel biomarkers and therapeutic targets. This review discusses the latest computational methods for inferring CCIs from different omics data and various CCI and drug databases, emphasizing their applications in predicting drug responses. We also comprehensively summarize recent patents, clinical trials, and publications that leverage these cellular interactions to refine cancer treatment approaches. We believe that the integration of these CCI-focused technologies can improve personalized therapy for cancer patients, thereby optimizing treatment outcomes and paving the way for next-generation precision oncology.

## Background

The tumor microenvironment (TME) is a dynamic cellular ecosystem composed of diverse cell types engaged in cell–cell interactions (CCI), both within and beyond the tissue milieu. A plethora of molecules mediate CCIs, and they may exist as intracellular, surface-bound, and/or secreted proteins or non-protein signals, for instance, transcription factors, cytokines and cytokine receptors, and ionic messengers [1]. These interactions play a crucial role in both maintaining the integrity and/or destruction of the TME. Thus, decoding how the TME shapes these responses is essential for the discovery of robust biomarkers and for the development of more effective therapies. However, the relationships between TME, CCI, and drug responses are multifaceted and complex, requiring comprehensive characterization and understanding.

In this review, we define drug response as any measurable changes in cancer cell behaviors such as alterations in proliferation, apoptosis, migration, or signaling pathways, which are triggered by a therapeutic agent. We also define measurement of CCI, which focuses on means to predict or quantify direct and/or indirect functional interactions, particularly those that produce downstream phenotypic effects. In our view, there are two major definitions of cellular interactions: (1) CCI defined by co-expression of specific ligands and receptors; and (2) spatially defined cell–cell colocalization (CCC) due to cells and/or markers being physically co-located in the same region, which may or may not represent specific ligand-receptor pairing. These two definitions are interconnected but are quantified/inferred differently, as we will discuss later.

### How CCI/CCC can be used for studying or predicting drug responses.

Recent advances in omics technologies now allow us to predict and measure CCI at varying depths of resolutions, from single-cell gene/protein expression of relevant molecules to spatially resolved gene, protein, and metabolic measurements.

**Fig. 1:**
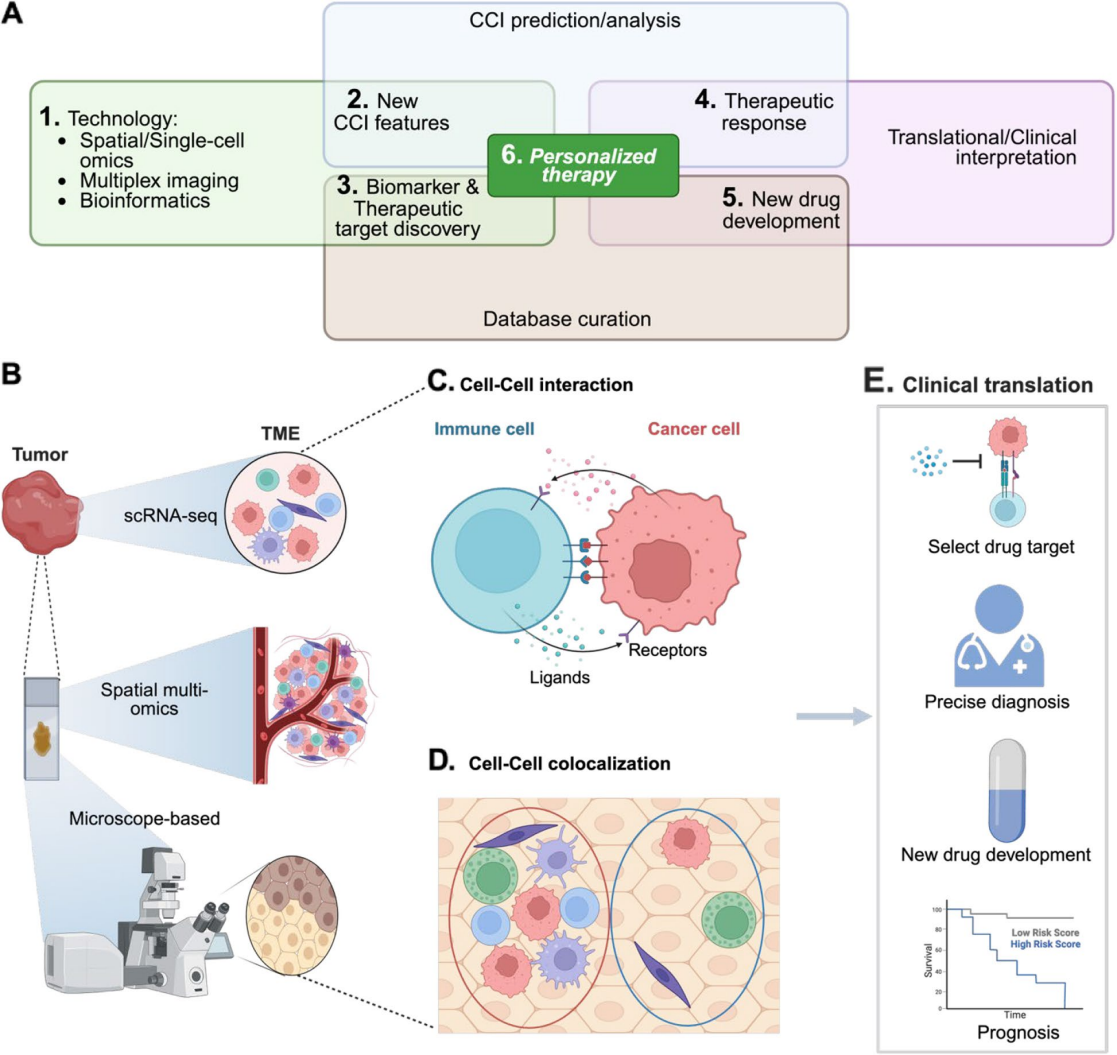
Schematic overview of an integrative approach using cell–cell interaction (CCI) features for personalized therapy in cancer treatment. **A** The technological foundation is spatial transcriptomics and single-cell RNA sequencing (scRNA-seq), which are used to analyze the tumor microenvironment (TME) (1), identifying new CCI features (2). These features feed into a database for curation and facilitate biomarker discovery (3). CCI analysis to identify previously discovered biomarkers is then used on patient samples to understand therapeutic response (4). Knowledge gained can then be used to inform on new drug development (5). Findings can then be integrated with patient's clinical and omics profiles to be used for personalized therapy (6). The diagram overlaps between the top sections, highlighting the interplay required to achieve clinical translation and personalized therapy. **B** Methods to detect TME. **C** Cell–cell interaction demonstrated by ligand-receptor interactions. **D** Cell–cell colocalization neighborhoods. Left panel: direct contact between cells; Right panel: cells without direct contact but in neighborhood. **E** Clinical translation: wherein patient response to therapy is assessed and utilized to predict long-term survival outcomes, as represented by the survival curve. This integrative approach exemplifies the translation of advanced scientific discoveries into personalized patient care. Figures were generated using BioRender.

Our objective is to provide readers with a comprehensive overview of the evolving understanding of CCI/CCC and how they can be harnessed for treating cancer (Fig. 1). We recognize the significance of CCI/CCC in influencing therapy sensitivity and resistance, particularly considering emerging immunotherapy approaches. For example, immunotherapies like immune checkpoint inhibitors (ICIs) modulate interactions between cancer cells and immune cells.

Traditionally, Hematoxylin and Eosin (H&E) staining, immunohistochemistry (IHC) and immunofluorescence (IF) are widely used diagnostic techniques in histopathology. IHC/IF staining for specific cancer-associated markers has been used to evaluate vascularization and immune cell infiltration with specific markers such as CD31 and F4/80. Multiplex-IHC/IF (mIHC/mIF) increases the multiplexing capacity of possible markers for comprehensive assessment and is highly reproducible and efficient.

However, these techniques require professional and competent pathologists to review many sets of images. Pathologist annotations, while very effective in diagnosing diseases, are qualitative and are particularly prone to both inter-observer and intra-observer variability when assessing feature-rich phenomena such as tumor-associated lymphocyte infiltration. In addition, existing methods utilize only histological information, so their usefulness in studying detailed structures in the TME is limited. Nevertheless, the use of artificial intelligence is now increasingly adopted in the clinic to help pathologists in performing their critical tasks [2].

High-throughput technologies enable the prediction of potentially millions of interactions between all cell types within a TME, allowing for individualized assessment of potential ligand-receptor (L-R) candidates as biomarkers and/or drug targets. These advancements can complement traditional H&E and IHC/IF staining to assess tissue architecture, cellular composition, and molecular profiles simultaneously within the same spatial context. They have been used effectively to study the progression, metastasis, and drug response of several cancer types, such as breast cancer [3, 4], head and neck cancer [5], and skin cancer [6]. For example, spatially resolved single-cell analysis (e.g., imaging mass cytometry (IMC)) revealed that the breast cancer TME architecture is composed of distinct tumor and stromal cell niches, which correlated with novel cancer subgroups and clinical outcomes [4]. Similarly, spatial-derived signatures were used to predict the likelihood of a patient being responsive to cancer immunotherapy in a patent application (US20220178926A1).

### Methods for inferring CCI/CCC and predicting drug responses

CCI analysis focuses on understanding the functional interplay between cells, assuming that cells are directly/indirectly interacting or communicating with each other locally due to physical co-location or over long distances with soluble molecules [7]. This interaction can occur through physical connections (like synapses in neurons) or via the release and receipt of soluble factors such as cytokines. In contrast, CCC analysis interprets whether two signal measurements co-distribute within a tissue or whether two cell types could predict to be physically collocated in close proximity without necessarily implying physical interaction. Colocalization can be thought of as consisting of two components: co-occurrence [8], which is the simple spatial overlap of two molecular measurements, and correlation, where two probes not only overlap but also co-distribute in proportion to one another within and between structures. Pearson's correlation coefficient serves as a useful statistic for quantifying colocalization [9]. An alternative yet closely related metric is the Manders Overlap Coefficient [9].

Several computational methods have been developed to predict CCI, specifically cell–cell L-R interactions, based on gene expression profiles and have been widely implemented in single-cell analysis (Table 1). The methods assume that cell-type pairs that express cognate L-R pairs are likely to interact with each other [10, 11]. One of the main advantages of this technique is that the technologies and tools needed for conducting scRNA-seq studies have become more accessible and efficient, making it a reliable source for the analysis of data [12]. In addition, there are now methods to sequence physically interacting cells (PIC-seq) [13], which directly isolate cell pairs that are physically interacting within a tissue using flow cytometry sorting or microfluidics, followed by sequencing.

**Table 1:** Popular computational CCI methods and integration of drug databases

Tools	Modality	Method type	Involves AI	Data type	Spatial dimension	Multi-omics integration	CCI databases	Human or mouse
CellPhoneDB [10, 27]	RNA	Permutation	N	scRNA-seq	Y	N	~ 1100 L-R pairs	Human
CellChat [11]	RNA	Rule-based mass-action	N	scRNA-seq	Y	N	~ 2000 L-R pairs	Both
ICELLNET [31]	RNA	Weighted scoring	N	scRNA-seq	Y	N	~ 2500 L-R pairs	Human
RNA-Magnet [32]	RNA	Rule-based	N	scRNA-seq	Y	N	~ 700 L-R pairs	Mouse
NATMI [33]	RNA	Statistical network	N	scRNA/Bulk-seq	Y	N	ConnectomeDB	21 different species
spaOTsc [34]	RNA	Machine learning	Random forest + optimal transport	ST	Y	N	~ 1000 L-R pairs	Both
SingleCellSignalR [29]	RNA/Protein	Ranking	N	scRNA-seq/single-cell proteomics (CyTOF and SCoPE-MS)	Y	N	~ 3200 L-R pairs	Both
NCEM [35]	RNA	Deep learning	GNN	scRNA-seq/ST	Y	Y	N	Both
SpaTalk [36]	RNA	Deep learning	Knowledge-graph GNN	ST	Y	N	KEGG and Reactome	Both
stLearn [18]	RNA	Permutation	N	ST	Y	N	ConnectomeDB	Both
scTensor [30]	RNA	Machine learning	Tensor decomposition	scRNA-seq	Y	N	~ 34,000 L-R pairs/ Connectivity Map	Both
SpotSC [37]	RNA	Probability model	N	scRNA-seq	Y	N	N	Both
CellTalkDB [38]	RNA	Database	N	scRNA-seq	Y	N	3398 human L-R pairs and 2033 mouse L-R pairs	Both
iTALK [39]	RNA	Ranking	N	scRNA-seq	Y	N	~ 2600 L-R pairs	Both
Connectome [40]	RNA	Network	N	scRNA-seq	Y	N	~ 2200 L-R pairs	Both
COMMOT [41]	RNA	Deep learning	Optimal transport + GNN	ST	Y	Y	CellChatD (L-R database), scSeqComm (downstream target genes); TF_TG_TRRUSTv2 and TF_TG_TRRUSTv2_RegNetwork_High_mouse were used for human and mouse Target gene, respectively	Both
scMLnet [42]	RNA	Machine learning	Multilayer network regression	ST	Y	N	LigRecDB (L-R database), RecTFDB (Receptor-TF database) and TFTGDB (TF-Target Genes database)	Both
MISTy [26]	RNA/Protein	Machine learning	Random forest	Imaging mass cytometry and ST	Y	N	No L-R databases Used cell type marker gene	Not species-specific
HoloNet [43]	RNA	Deep learning	Graph attention network	ST and scRNA-seq	Y	Y	CellChatDB database	Both
CytoTalk [44]	RNA	Machine learning	Prize-collecting Steiner forest	ST and scRNA-seq	Y	Y	Reactome, KEGG, WikiPathways	Both
NicheNet [45]	RNA	Machine learning	Elastic-net regression	scRNA-seq	N	N	L-R interactions: KEGG, IUPHAR; Predicted L-R interactions: Omnipath, PathwayCommons, InWeb_InBioMap, ConsensusPathDB, EVEX, KEA, PhosphoSite, DEPOD; Gene regulatory interactions: RegNetwork, TRRUST, HTRIDB, ReMap, EVEX, PathwayCommons, Ontogenet, CHEA, ENCODE, JASPAR, TRANSFAC, MOTIFMAP, GEO, and MSigDB	Both
Scriabin [46]	RNA	Deep learning	Graph attention network	ST and scRNA-seq	Y	Y	CellPhoneDB and OmniPath	Both
proximID [47]	RNA	Experimental mapping	N	scRNA-seq	Y	N	No L-R DB	Mouse
CSOmap [48]	RNA	Optimisation	N	scRNA-seq / IHC	Y	N	FANTOM5	Both
SVCA [49]	RNA/Protein	Variance components	N	IMC/ST	Y	N	No L-R DB	Both
GCNG [22]	RNA	Deep learning	Graph convolutional network	ST	Y	N	No L-R DB	Mouse
MESSI [50]	RNA	Deep learning	Mixture-of-Experts neural network	ST	Y	N	No L-R DB	Mouse
Tensor-cell2cell [51]	RNA	Machine learning	Tensor decomposition	scRNA-seq	N	N	CellChatDB	Both
LIANA + [52]	RNA	Permutations	N	scRNA-seq / ST	Y	Y	OmniPath, etc	Both
spatialDM [53]	RNA	Bivariant Moran’s statistic	N	ST	Y	N	No L-R DB	Both
DeepLinc [54]	RNA	Deep learning	Variational graph autoencoder	ST	Y	N	No L-R DB	Both
SpaCI [55]	RNA	Deep learning	GNN	ST	Y	Y	No L-R DB	Both
imcRtools [56]	Protein	Workflow	N	IMC	Y	Y	No L-R DB	Both
Giotto [20]	RNA/Protein	Statistical, Workflow	N	scRNA-seq /ST/SP	Y	Y	FANTOM5	Both
C-SIDE [57]	RNA	Permutations	N	ST	Y	Y	No L-R DB	Both
stMLnet [42]	RNA	Machine learning	Tree-based	ST	Y	Y	LigRecDB, etc	Both

Spatial transcriptomics is another emerging approach that provides spatial context to CCI/CCC analysis. Spatial transcriptomics technologies can preserve the spatial context of cells, allowing researchers to understand how cancer cells interact with neighboring non-cancerous cells and how they contribute to cancer progression [14]. Moreover, tumors are heterogeneous with highly variable TME dynamics within each spatial niche. Several software packages such as SpatialDE [15] and Spark-X [16] have implemented regression models for spatially co-expressed genes. Squidpy [17] and stLearn [18] apply spatial autocorrelation tests with metrics such as Moran’s *I* and Geary’s *C* [19]. Giotto [20] focuses on spatial correlation, while histoCAT [21] and stLearn [18] leverage spatial information to define cell neighborhoods and implement two levels of permutation testing, for both genes and cells/spots, to identify spatial locations where a given L-R pair exhibits significantly higher interaction scores than expected by chance. This approach helps to mitigate the false discovery rate, which is a common issue in non-spatial CCI analysis, thus enhancing the reliability of the findings [15]. There are also approaches to predict extracellular interactions from spatial transcriptomics, such as Giotto (unsupervised correlation-based) [20] and graph convolutional neural networks (GCN) (deep-learning based) [22].

Spatial proteomics have significant potential applications in the clinical setting and are becoming more automated, accessible, and affordable, and have the potential to identify markers missed by traditional bulk proteomic methods. For example, mass spectrometry-based spatial proteomic approaches have been used to quantify metabolites and proteins directly, surpassing traditional methods in sensitivity, specificity, and the ability to measure a broader range of analytes [23]. In addition, mIF-based spatial proteomics, such as PhenoCycler from Akoya, allows for the simultaneous detection and quantification of hundreds of proteins within a single tissue sample, providing subcellular spatial information about protein localization and abundance [24], and is increasingly adopted in clinical histopathology. To infer CCIs from spatial proteomics data, several dedicated pipelines have been developed. For example, histoCAT applies spatial permutation testing on protein co‐expression to identify statistically enriched L-R neighborhoods [21]. SPIAT (Spatial Image Analysis of Tissues) computes proximity networks and neighborhood enrichment of cell-type pairs directly from mIF or multiplexed ion beam imaging (MIBI) images [25]. Additionally, MISTy has also been adapted to integrate spatial proteomic measurements by modeling intra- and intercellular signaling through multi-view learning [26]. Incorporating spatial coordinates of cells with their L-R expression improves the inference of CCIs from spatial omics data by ensuring that putative communication events are physically plausible and context-specific. Advanced spatial technology platforms now achieve single-cell spatial resolution and measure dozens of proteins or thousands of transcripts per tissue (e.g., Xenium, STomics, and PhenoCycler), yielding rich datasets of cell locations and phenotypes. Computational tools leverage these data by integrating spatial adjacency with known L-R pairs: a common strategy is to construct spatial cell graphs linking neighboring cells and then evaluate L-R co-expression across these contacts. For example, Giotto uses spatial location information to build a spatial proximity graph to identify L-R interactions [20]. stLearn further not only considers spatial location but also takes into account spatial cell-type heterogeneity of L-R co-expression [18]. Spatially aware CCI inference can reveal communication patterns in situ that would be missed by non-spatial analyses. Incorporating spatial constraints can also reduce false positives by filtering out interactions between distant cells.

Most of the current tools were created for general CCI/CCC analysis for single-cell and/or spatial data. We list some of the more popular CCI/CCC packages specifically on whether these tools have integrated information from drug databases, summarizing this in (Table 1). We note that most of them contain ligand and receptor pairs that can also be found in popular drug databases. For example, drug2cell integrates CellPhoneDB [10, 27] with the ChEMBL database to identify high-confidence drug-target associations, filtering these associations by potency and clinical approval status, and overlays the resulting targets onto cell-type-resolved L-R pairs so that potential pharmacological modulators of each interaction can be prioritized [28]. Similarly, scTensor provides an L-R database resource (selected for the relevant species, e.g., CellPhoneDB and SingleCellSignalR [29] database for human) that is periodically linked to Connectivity Map (CMap) perturbation signatures, enabling every inferred L-R pair to be cross-referenced with transcriptional responses to thousands of small molecules and genetic perturbagens [30].

### Drug databases useful for CCI and CCC

**Fig. 2:**
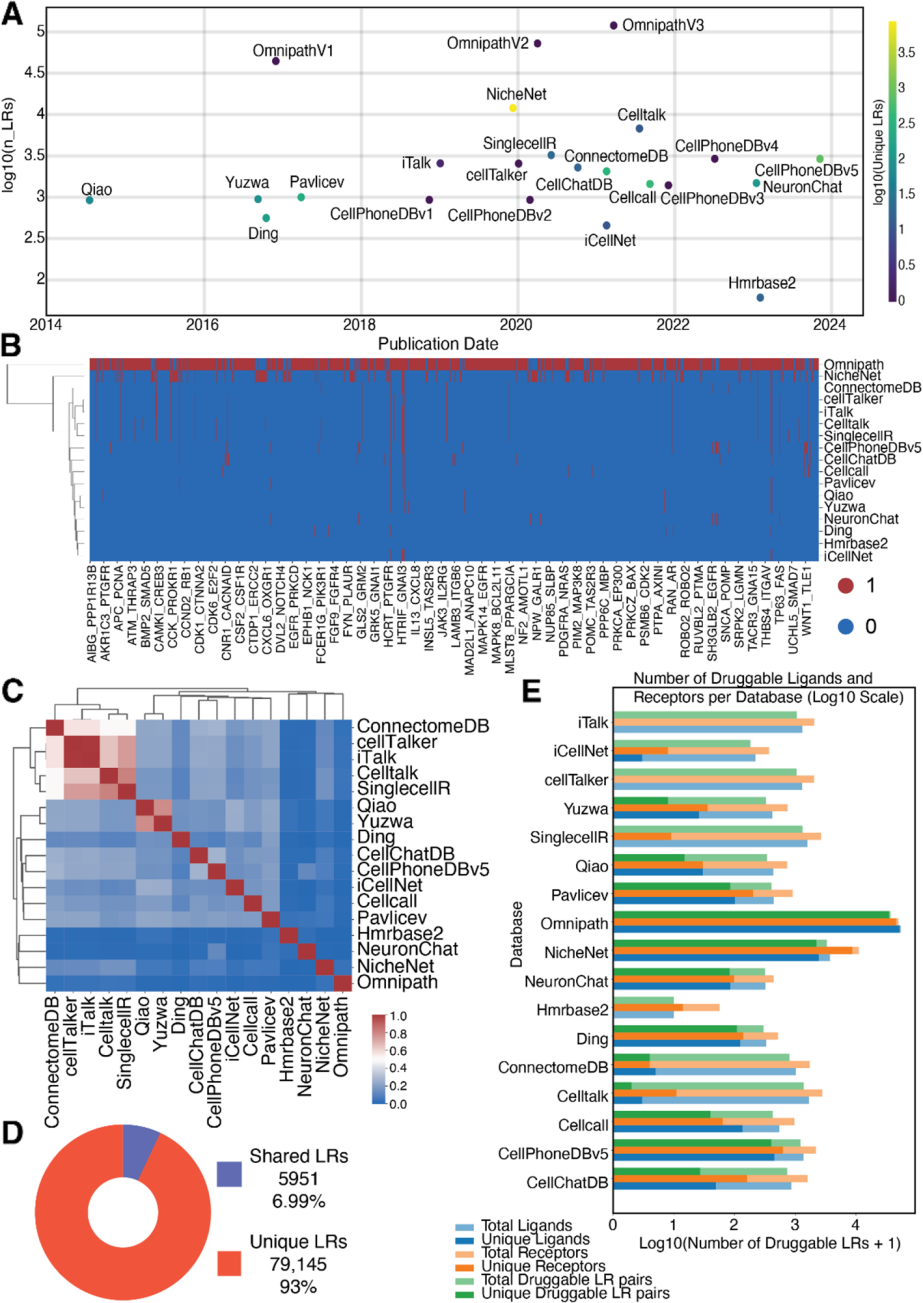
L-R databases used for CCI analysis. **A** The CCI database we collected and their publication date. **B** The heatmap shows the presence of L-Rs in each database. **C** The heatmap shows the similarity of each database. **D** The pie chart shows the number/proportion of L-Rs shared across different databases. **E** Number of ligands (blue), receptors (orange), and L-R pairs (green), also known as drug target genes in the ChEMBL database. Light colors indicate the proportion of shared ligands, receptors, or pairs across different L-R databases. Solid colors indicate those unique to each database

Study of CCI in the context of identifying therapeutic targets is supported by a wealth of L-R databases (Table 2). Our analysis reveals that there are approximately 85 K recorded interactions across various CCI databases (Fig. 2A–B). OmniPath [58] is the most comprehensive database, integrating data from over 100 resources (Fig. 2B). Further comparative evaluation highlights notable discrepancies and overlaps among these databases (Fig. 2C–D). We found that different CCI databases have their own unique L-Rs, while some databases are very similar (e.g., CellTalkDB [38] and iTalk [39]) (Fig. 2C). Overall, we found that 93% of L-Rs are unique, while 7% of L-Rs are shared across all databases (Fig. 2D).

**Table 2:** Databases for predicting druggable CCI

Name of database	Website link
Database of ligand-receptor interaction	
connectomeDB2020	https://asrhou.github.io/NATMI/
Druggable targets or gene/drug interaction	
DrugBank	https://go.drugbank.com/ (Needs payment)
Therapeutic Target Database	https://db.idrblab.net/ttd/
Cancer Drug Database	https://www.anticancerfund.org/en/cancerdrugs-db
IUPHAR/BPS	https://www.guidetopharmacology.org/
DGIdb	https://www.dgidb.org/
Drug2cell	https://www.sanger.ac.uk/technology/drug2cell/
Repurposing Data Portal	https://repo-hub.broadinstitute.org/repurposing-app
Drug sensitivity/resistance information	
DRESIS	https://dresis.idrblab.net/
Genomics of Drug Sensitivity in cancer (GDSC)	https://www.cancerrxgene.org/
Cancer Therapeutics Response Portal (CTRPv2)	https://portals.broadinstitute.org/ctrp.v2.1/?page=#ctd2BodyHome
Cellular Drug Response Atlas (CeDR)	https://academic.oup.com/nar/article/50/D1/D1164/6389514
Potential drug development	
BindingDB	http://bdb2.ucsd.edu/bind/index.jsp
ChEMBL	https://www.ebi.ac.uk/chembl/
Harmonizome	https://maayanlab.cloud/Harmonizome/
Cell/spatial databases for drug target evaluation	
Human Tumor Atlas Network (HTAN)	https://humantumoratlas.org/
Human Cell Atlas (HCA)	https://www.humancellatlas.org/
Human BioMolecular Atlas Program (HuBMAP)	https://hubmapconsortium.org/infrastructure/
Human Protein Atlas (HPA)	https://www.proteinatlas.org/
Comprehensive Repository Of Spatial Transcriptomics (CROST)	https://ngdc.cncb.ac.cn/crost/home
Spatial omics resource of Cancer (SORC)	http://bio-bigdata.hrbmu.edu.cn/SORC/index.jsp
Spatial transcriptOmics Analysis Resource (SOAR)	https://soar.fsm.northwestern.edu/
Packages/Tools using single-cell spatial dataset for drug responses/personalized treatment	
SpaRx	https://github.com/QSong-github/SpaRx

Given that many ligands and receptors serve as targets for approved or investigational drugs, bridging CCI resources with drug target databases should help translate the interaction maps into therapeutic hypotheses, including for flagging novel interactions for compound screening and/or drug repurposing.

For example, there are databases that include information on druggable targets, such as DrugBank [59], The Therapeutic Target Database (TTD) [60], and the Cancer Drug Database [61]. For drug repurposing, the Repurposing Data Portal [62] is a large and comprehensive resource that offers new possibilities for applying existing drugs to cancer treatment. This is particularly relevant in the context of a pan-cancer assessment of common L-R interactions and the expression of drug targets, which suggests that some drugs may be effective across multiple cancer types. In the field of drug sensitivity and resistance, DRESIS [63], the Genomics of Drug Sensitivity in Cancer (GDSC) [64], and the Cancer Therapeutics Response Portal (CTRPv2) [65, 66] provide invaluable data for understanding the variations in drug responses across different cancer types. Lastly, BindingDB [67] and ChEMBL [68] offer extensive information on drug-binding activities and molecular bioactivity, which is crucial for novel drug development.

For the evaluation of drug candidates, databases that provide spatial and cellular data, such as Human Tumor Atlas Network (HTAN) [69], the Human Cell Atlas (HCA), the Human BioMolecular Atlas Program (HuBMAP) [70], Human Protein Atlas (HPA) [71], the Comprehensive Repository of Spatial Transcriptomics (CROST) [72], and the Spatial Omics Resource of Cancer (SORC) [73] can be leveraged not only to contextualize drug responses across diverse cell types and tissues, but also to gain insights into mechanisms of action.

Overall, these databases collectively form a comprehensive network of resources that can support research into drug development for CCI targets.

## Case studies on CCI and CCC as markers for drug responses

CCIs/CCCs are interesting avenues for understanding drug responses and enhancing cancer treatment. The challenge in the translational setting is to effectively integrate the complexity of CCIs/CCCs into predictive and therapeutic strategies. Here, we will highlight some recent patents, clinical trials, and publications that apply single-cell or spatial technologies to investigate CCI/CCC in cancer drug responses.

### Innovation in harnessing CCIs and CCCs for cancer therapy

We selected patents that illustrate recent technological developments at the intersection of single-cell/spatial-omics and personalized cancer treatment. The patents presented in Table 3 are not an exhaustive list, but rather a curated selection of notable innovations identified through a targeted search on Google Patents using keywords such as “cell–cell interaction,” “cell–cell colocalization,” “precision medicine,” “cancer,” “spatial omics,” and “scRNA-seq.” For instance, one patent (US20230016003A1) discussed an invention that involves labeling cancer tissue samples with molecular probes and using multiplexed imaging to assay the expression of various biomolecules at a single-cell level. Based on the expression patterns, breast cancer patients could be categorized into 18 “Single Cell Pathology” groups. Each group is linked with a favorable/unfavorable response to anti-neoplastic drugs and/or survival outcome, thus allowing the inventors to stratify patients according to the desired therapy response and outcome.

**Table 3:** Patents using single-cell or spatial technology to investigate cell–cell interaction in oncology

Patent	Method	Indication (application)	How CCI is being used	Innovation
"Single cell pathology analysis of tumour samples" (US20230016003A1)	(1) A method involving labeling cancer samples with molecular probes to assay the expression of various biomolecules at a single-cell level (2) Based on these expression patterns, a Cellular Identity is assigned to each cell, and patients are categorized into Single Cell Pathology groups	Advantageous for patients with hormone receptor-expressing cancers like breast and ovarian cancer	By quantifying the composition and co-occurrence of different cell identities, it derives community-level CCI metrics that stratify patients into “Single-Cell Pathology” groups for prognosis and therapy guidance	This analysis aids in tailoring anticancer treatment plans based on the Single Cell Pathology group assignments
“Method for predicting patient response to immunotherapy” (US20230065757A1)	A method to predict how a patient would respond to immunotherapy, particularly focusing on Cutaneous T cell lymphoma (CTCL) The method comprises performing a multiplexed binding assay on patient’s tissue section to identify cancer cells, effector immune cells and immunosuppressive cells; For each effector T cell, the physical distances to its nearest tumor cell and its nearest immunosuppressive cell are measured and their ratio calculated	Stratify patient into probable responders and non-responders to immunotherapy like pembrolizumab before therapy initiation, leading to more personalized and effective treatment strategies for CTCL patients	Spatial CCIs are quantified by comparing immune–tumor and immune–suppressor cell proximities. The resulting SpatialScore reflects functional interactions that predict therapeutic response	Calculation of a ratio-based SpatialScore predictive of immunotherapy outcomes
“A method for determining the likelihood of a patient being responsive to cancer immunotherapy” (US20220178926A1)	Identifying and quantifying tumor‐infiltrating T cells from ER + breast cancer that express markers such as PD-1, CTLA-4, CD38, CD45, CD3, CD25, TIM-3, FOXP3, CD4, and CD8 If certain ratios of these markers are above a threshold, the patient is considered to have a high likelihood of responding to cancer immunotherapy	Predict the likelihood of a patient's responsiveness to cancer immunotherapy	Measuring co-inhibitory and co-stimulatory receptors on T cell subsets as surrogate metrics for T cell–tumor cell crosstalk and immune suppression	Assigning a high likelihood of responsiveness if certain ratios of marker expressions are above defined thresholds
“Methods of determining a surgical margin and methods of use thereof” (US11781130B2)	Advanced methods for determining surgical margins in cancer tissue resection. The patent uses a specialized array of spatially barcoded probes that bind to specific analytes in different tissue locations. By comparing analyte presence across various tissue locations, the method aids in accurately identifying surgical margins, determining the size and site of tissue resection, and reducing the risk of re-excision and recurrence of tissue abnormalities	This approach represents a significant advancement in precision surgery, offering a more targeted and effective method for tissue resection, especially in oncological surgeries	Spatial CCC is inferred by mapping where tumor specific analytes and surrounding stromal or immune cell markers co-occur or abut. These local neighborhood patterns identify functional interaction zones at the invasive front, guiding margin definition	Using spatial technology to find tumor margin during surgery
“Multimodal fusion for diagnosis, prognosis, and therapeutic response prediction” (US20220367053A1)	A deep‐learning algorithm employs tensor fusion to provide end-to-end multimodal fusion to model the pairwise interactions of features across multiple modalities (e.g., histology and molecular features)	Enhanced diagnosing, prognosticating, and predicting therapeutic responses in cancer treatment by integrative analysis of phenotypic and genotypic tumor features	By explicitly modeling interactions between spatial histological contexts (cell neighborhoods, tissue architecture) and molecular expression profiles (L-R signals, cellular phenotypes), the tensor‐fusion approach captures both spatial CCC and inferred functional CCI patterns as predictive features	The fusion of different types of data using a deep learning-based algorithm, including morphological information from histology and molecular information from omics This algorithm employs tensor fusion to model interactions of features across multiple modalities

Another patent (US20230065757A1) describes an invention based on multiplexed binding assays for predicting patient responses to the PD-1-blocking antibody pembrolizumab, with a focus on cutaneous T cell lymphoma. This assay identifies various cell types, including cancer cells, effector immune cells, and immunosuppressive cells, and the primary aim is to stratify patients into categories of probable responders and non-responders to immunotherapy treatments like pembrolizumab to facilitate more personalized and effective treatment strategies.

Similarly, another patent (US20220178926A1) uses mass cytometry to quantify the expression of specific immune/tumor cell CCI markers and to quantify immune/tumor cell proportions and ratios in breast cancer. If certain ratios of these markers are above a threshold, the patient is considered to have a high likelihood of responding to ICI therapy.

Patents involving deep learning have also been described (US20220367053A1), where different types of medical information, including images from scans and genetic data, are combined using the deep learning program to get a better understanding of a patient's cancer status, giving a comprehensive view of multiple facets of the patient’s disease, which can lead to better treatment decisions. This approach is part of a larger trend in medicine to use technology to integrate and analyze different kinds of health data for better patient care.

Overall, these patents support CCIs and CCCs as useful biomarkers that may improve drug development pipelines, particularly in tracking and predicting therapy responses and stratifying patients for more personalized cancer treatments. Each of these patents not only describes the means to measure the CCI/CCC but also incorporates new algorithms and applications, highlighting the need for these components to work in concert to advance the technological capabilities and translate complex cellular interactions into actionable clinical insights for tumor therapy.

### CCI and CCC profiling in Clinical trials

Several clinical trials (Table 4) have focused on targeting CCIs in cancer progression for their treatment strategies over a diverse range of cancer types such as nasopharyngeal carcinoma, rhabdomyosarcoma, triple-negative breast cancer, medulloblastomas, liver metastases, and different stages of breast cancer. The treatment modalities explored in these trials are also diverse, including chemotherapy, radiation, neoadjuvant therapies, and immunotherapy. These studies all focus on biomarkers, diagnosis, prognosis, and understanding patient-specific responses and resistance to treatments.

**Table 4:** Clinical trials using single-cell or spatial technology to investigate cell–cell interaction in oncology

NCT Identifier	Trial phase	Disease	Stage	Drug	Mechanisms	Biomarkers	Diagnosis	Prognosis	Responses	Resistance	Samples	Repeat	Country	Sample Size	Reference
NCT02977195	1	Advanced solid tumor; endometrial carcinoma, hormone receptor positive tumors	Advanced stage	Anti-netrin-1 antibody NP137	x			x	x				France	42	[3, 74]
NCT03299946	1	Hepatocellular carcinoma	Locally Advanced	Neoadjuvant Cabozantinib Plus Nivolumab	x				x		Surgical resections		US	15	[77]
NCT05912582	4a	Nasopharyngeal carcinoma	Stage IVa	Chemo and radiation				x	x		Tumor, blood	X	China	60	-
NCT06040229	2	Rhabdomyosarcoma	Recurrence	NA	x					x	Tumor	No	China	6	-
NCT05910710	3	Triple-negative breast cancer (TNBC)	NA	Neoadjuvant chemotherapies	x	x		x	x			No	Korea	50	-
NCT05672043	1	Medulloblastomas		NA				x			Tumor, blood	No	China	400	-
NCT04622423	?	Liver metastasis	IV	NA	x	x		x			Primary and metastatic tumor, blood	x	Italy	475	[78]
NCT04631731	1/2	Liver, gastrointestinal, endocrine and skin toxicity	III-IV	PD-1i, PD-L1i, CTLA4i, Tyrosine Kinase inhibitor, VEGFi	x		x		x	x	Tumor, blood	x	Australia	200	[76]
NCT05912062	3	HER + breast cancer	Early	antiHER2				x	x					80	-
NCT02657889	1/2	TNBC and ovarian cancer	Advanced	Niraparib + Pembrolizumab	x			x			Tumor and blood		USA	62	[75]

For example, trial NCT02977195 demonstrated how anti-netrin-1 antibody NP137 influences lung metastasis [3, 74]. Visium analysis of pre- and post-treatment biopsies from two patients showed that NP137 reduced tumor cell proportions linked with a profound shift in gene expression related to tumor epithelial-mesenchymal transition. There were changes in immune infiltrate and increased interactions between cancer cells and the TME. Another Phase 1/2 study (NCT02657889) highlighted the interactions between CD8 + T-cells, PD-L1 + macrophages, and the efficacy of combining PARP and PD-1 inhibitors in treating breast and ovarian cancer [75].

Another trial with a larger sample size in Australia used Visium to analyze baseline and post-Ipilimumab and Nivolumab therapy samples from a cohort of malignant pleural mesothelioma patients (NCT04631731) [76]. They analyzed tumor-specific immune cell gene signatures and found differences in T cell distribution and gene expression patterns between patients who did or did not develop immune-related adverse events. These studies highlight the utility of CCI/CCC analysis as research tools with clinically informative insights into therapy mechanisms that correlate with patient outcomes. The application space for CCI/CCC profiling is clearly diverse, and these approaches are now at the stage where they are revealing substantial new knowledge about tumor and immune biology and interaction, which can serve as the foundation for future personalized therapeutic developments and clinical applications.

### Using CCI and CCC to understand therapy responses

There are numerous publications that have investigated the utility of targeting CCIs in the evolving landscape of precision oncology. For instance, in ovarian cancer, epithelial-stromal crosstalk, particularly TGF-β signaling in cancer-associated fibroblasts (CAFs), has been correlated with poor patient survival, and these signaling networks can potentially represent novel druggable targets [79].

Single-cell spatial architectures have been correlated with clinical outcomes in head and neck squamous cell carcinoma (HNSCC), where the presence/absence of mesenchymal cellular neighborhoods was associated with patient survival [80]. Similarly, in TNBC, MIBI linked TME spatial organization with survival rates, highlighting the heterogeneity and complexity of tumor-immune interactions [81].

In the context of immunotherapy, spatial analysis has highlighted that the proximity of L-R expression (e.g., PD-1/PD-L1), the spatial distribution of immune and tumor cells, and/or cellular neighborhoods/niches are linked to treatment responsiveness/effectiveness [82–85].

These studies highlight the utility of CCI/CCC analysis as research and clinically informative tools for understanding therapy mechanisms and patient outcomes. The application space for CCI/CCC profiling is clearly diverse, and these approaches are now at the stage where they are revealing substantial new knowledge about tumor biology, which can serve as the foundation for future personalized therapeutic developments and clinical applications (Table 5).

**Table 5:** Publications using single-cell or spatial technology to investigate CCI in oncology

PMID	Context	Indication	Innovation	
37704757	Integrated spatial transcriptomics (Visium) and proteomics (PhenoCycler) data in a patient of oropharyngeal squamous cell carcinomas who failed Nivolumab and Pembrolizumab/Lenvatinib therapies to explain rationale for treatment failure and explore alternative therapeutic targets	Discovery and ranking of clinically relevant alternative medicines based on ligand-receptor interaction within tumor region Indicating the potential mechanism for treatment failure	Developing SpiCi (Spatial Proteomics inferred Cell identification) which can resolve the profiling of tumor infiltrating immune cells	
37153589	Identifying PD-L1, B7-H3, and VISTA and tumor necrosis factor receptor superfamily members as biomarkers of response to Pembrolizumab or Nivolumab	Support the role of immune checkpoint molecules and implicates the TNFR superfamily as key players in immunotherapy response in our cohort of HNSCC	Demonstrating how targeted spatial proteomic approaches may provide new cues to identify biomarkers of ICI therapy	
29860390	Finding potential therapeutic targets in ovarian cancer	Activation of TGF-β-dependent and TGF-β-independent Smad signaling was identified in a particular subtype of CAFs and was associated with poor patient survival	The methodical identification of these epithelial-stromal crosstalk signaling networks, which could potentially lead to the discovery of new drug targets, thus broadening the scope of ovarian cancer treatment	
35217711	Correlate single-cell spatial architectures with clinical outcomes in HNSCC	Mesenchymal (αSMA +) cellular neighborhoods describe distinct immune landscapes associated with neoplastic tumor-immune compartmentalization and improved patient outcomes	Unveiling associations between spatial cellular configurations and clinical outcomes, potentially informing prognosis and treatment strategies in HNSCC	
30285852	Evaluating the correlation between PD-1/PD-L1 expression and responsiveness to pembrolizumab treatment	PD-1/PD-L1 proximity was associated with clinical response, but CD8/PD-L1 proximity was not	Utilizing a multidimensional, quantitative approach to assess PD-1/PD-L1 expression, aiming to better understand and predict treatment responses to pembrolizumab	
32002281	Investigating anti-PD-1-based therapies' effectiveness in metastatic melanoma, focusing on immune and tumor cells' spatial distribution	In multivariate analysis, the best model for 12-month progression-free survival for anti-PD-1 monotherapy included PD-L1 + cells within proximity to tumor cells and intratumoral CD8 + density, and for combination therapy included CD8 + cells in proximity to tumor cells, intratumoral PD-L1 + density and LDH	Understanding interspatial distribution may predict therapy response, potentially improving patient outcomes	
29409671	Increased infiltration of regulatory T cells into core tumor regions is an independent predictor of worse overall survival in NSCLC Increased infiltration of CD8 + cytotoxic T cells among regulatory T cells mitigated this effect	G-cross spatial distance distribution method, which computes the probability of finding at least one immune cell of any given type within a defined radius of a tumor cell	G-cross analysis framework can be easily applied once staining has been performed as it uses the spatial information of different cell types as input, regardless of staining or imaging technique	
31194225	Interactions of exhausted CD8 + T-cells and PD-L1 + macrophages and PD-L1 + tumor cells as mechanistic determinants of response Defective homologous recombination DNA repair mutations also correlate with good response	Phase I/II clinical trial of the PARPi Niraparib in combination with the anti-PD-1 antibody Pembrolizumab in recurrent ovarian cancer (TOPACIO trial)	They show that mutational signature 3 and interferon signaling in the CD8 + T-cell compartment of the TME determine responses to Niraparib plus Pembrolizumab in patients enrolled in the TOPACIO trial	
32763154	Enrichment of PD-1 + CD4 + T cells only within granulocyte cellular neighborhoods positively correlated with survival in a high-risk patient subset	Re-engineering of co-detection by indexing (CODEX, currently commercialized as PhenoCycler) for paraffin-embedded tissue microarrays, enabling simultaneous profiling of 140 tissue regions from 35 advanced-stage colorectal cancer (CRC) patients with 56 protein markers	FFPE-optimized CODEX is suitable for highly multiplexed single-cell marker visualization, quantification and biomarker discovery in clinically relevant tissues	
30193111	Used multiplexed ion beam imaging by time-of-flight (MIBI-TOF) to simultaneously quantify in situ expression of 36 proteins covering identity, function, and immune regulation at subcellular resolution in 41 TNBC patients	Ordered immune structures along the tumor-immune border were associated with compartmentalization and linked to survival	Provide deeper insights into the structured TME, revealing variability across individuals, and linking spatial organization to survival rates	
36917954	Examining tumor cell PD-L1 (tPD-L1) function in tumor immune evasion	Tumor cell PD-L1 does not promote tumor immune evasion in the primary tumor site but engages myeloid cell PD-1 to activate SHP2 to antagonize the IFN-I-STAT1-CXCL9 intrinsic pathway in myeloid cells to suppress T cell recruitment in lung metastases	Identifying the mechanism where tPD-L1 engages myeloid PD-1 to activate SHP2, thus antagonizing the IFN-I and STAT1 pathway to impair cytotoxic T lymphocytes recruitment to lung metastases, providing insights for potential therapeutic interventions	

## Outlook

CCI/CCC data are increasingly integrated as prognostic indicators of disease severity and as biomarkers for assessing the response to various drug types, most notably ICIs, in multiple clinical settings. Artificial Intelligence (AI) can also rapidly process vast amounts of CCI/CCC data [86–88], identifying meaningful patterns to deeply analyze the complex interactions between cells. This capability helps in more accurately predicting the progression of specific diseases and the response to ICI treatment. Additionally, AI can be used to segment and stratify diverse patient groups, help predict disease severity, and suggest potentially safer, more cost-effective, and personalized treatment options [89–91]. For example, GraphComm employs a graph-based deep learning architecture, combining Graph Attention Networks (GATs) and Node2Vec embeddings to infer CCC using transcriptomic data and curated databases such as OmniPath [92]. It accounts for complex network structures and protein–protein interaction contexts and has been applied in drug perturbation settings to detect CCC changes in treated cancer cell lines, supporting its relevance to therapeutic prediction [92]. TxGNN is a graph foundation model for mapping transcriptomic signatures to drug response, trained on single-cell perturbation profiles. While this work provides a powerful strategy for predicting drug sensitivity, it does not incorporate CCIs/CCCs or microenvironmental context into its predictions [93]. Incorporating CCIs and CCCs derived from spatial omics data would enable the model to capture microenvironment-mediated resistance mechanisms that influence drug response, thereby improving the accuracy of drug-sensitivity predictions in heterogeneous tumor ecosystems.

To our knowledge, no method has explicitly integrated spatial transcriptomics or spatial proteomics data to explore CCIs/CCCs in the context of drug response to a large patient cohort. Our previous work deeply investigated one patient through the treatment journey, using PhenoCycler and Visium data to understand different responses at two rounds of treatment. We ranked L-R interaction in the immune hot tumor cores of the patient for the biopsies at the first treatment, when the immunotherapy (anti PD-1) failed [5]. Our CCI analysis results suggested alternative druggable targets could have been more effective for this patient. This was a proof of concept about the utilities of spatial multiomics to understand drug responses [5]. Given the physical constraints of spatial proximity, integrating spatial information could provide more precise and clinically relevant insights for identifying actionable drug targets.

High-throughput screening has demonstrated clinical benefits by enabling targeted drug treatments. For example, genetic testing for mutations of known drug targets, such as EGFR mutation screening for EGFR-tyrosine kinase inhibitors (through sequencing) or PD1/PD-L1 staining for immunotherapy (through imaging), is now the current standard of care. This provides a strong rationale for the development of assays with more comprehensive sequencing and high-throughput imaging to cover biomarkers of drug responses, opening a new era for “next generation pathology” where a diagnosis does not only rely on visible morphological features but also novel features such as interactions between cancer cells and surrounding cells within the microenvironments. Such predictions can bring about new abilities in pathology tests to predict drug responses. An active area of development is to predict gene expression from H&E images; for example, whether predicted CCIs are active in a patient so as to prioritize the drugs targeting the most active CCIs at the point of diagnosis.

### Evaluation and Validation

It is critical to validate CCI/CCC findings to translate into druggable targets. One important technique in studying and validating these interactions is the Proximity Ligation Assay (PLA), which allows the visualization of molecular interactions at the single-molecule level with high specificity and sensitivity [94]. In this assay, two proteins of interest are targeted with primary antibodies linked to oligonucleotide-conjugated secondary antibodies. If the target molecules are within 40 nm of each other, the oligonucleotides on the secondary antibodies undergo ligation, detectable by fluorescently labeled probes or rolling circle amplification [95]. Another emerging way of PLA is Labeling Immune Partnerships by SorTagging Intercellular Contacts (LIPSTIC) [96]. Specific cell surface proteins on two different cells are genetically modified to express “bait” and “prey” proteins. When these cells come into close proximity, the 'bait' protein triggers a reaction that adds a label (like a biotin tag) to the “prey” protein; then the cells can be analyzed using methods like mass spectrometry or flow cytometry to identify which proteins were tagged, providing insights into the interacting proteins at the contact site. The universal version of LIPSTIC (uLIPSTIC) [97] expands on the original concept of LIPSTIC by allowing for a broader application without the need to know the specific proteins involved in the CCIs beforehand. Typically, an enzyme capable of catalyzing the transfer of a biotin molecule (or another chemical group) is genetically fused to a protein known to be located on the cell surface. Common enzymes used for this purpose include biotin ligase or peroxidase. When cells come into proximity, the enzyme activates and tags nearby proteins on the opposing cell's surface. After the interaction, the cells can be treated to isolate the labeled proteins, which are then typically identified and quantified using mass spectrometry. This process allows researchers to determine not only the identity of the proteins but also potentially their functional roles in the interaction.

### Limitations and challenges

Despite the rapid progress and promising potential of CCI/CCC analyses in cancer research and precision medicine, several limitations must be acknowledged.

Technological limitations remain at the forefront. First, the inference of L-R interactions from transcriptomic or proteomic data is often indirect and does not guarantee functional interaction, particularly in spatial transcriptomics where mRNA abundance may not reflect actual protein availability or interaction dynamics. Additionally, most current tools are based on static snapshots of tissue architecture, which fail to capture the dynamic and temporal aspects of CCI/CCC within the TME. Second, the resolution and sensitivity of current spatial omics platforms vary, and lowly expressed ligands or receptors may go undetected, leading to incomplete interaction maps. Moreover, the majority of spatial datasets, especially proteomics, are still limited to a few hundred genes or markers, restricting the scope of interactions that can be assessed. Another major limitation is the lack of standardized benchmarks or gold-standard datasets, which complicates the validation and comparison of computational tools designed to infer CCI or CCC.

From a drug development perspective, many L-R interactions inferred from omics data do not have known small-molecule or biologic modulators, limiting their immediate druggability. Even when candidate targets are identified, translating them into therapeutics requires extensive functional validation, safety profiling, and biomarker development, which are time- and resource-intensive. Moreover, the spatial context required to prioritize targets based on cell proximity or interaction strength is often lost in traditional high-throughput drug screening pipelines. There is also a lack of robust frameworks to systematically incorporate CCI-derived biomarkers into existing clinical trial designs or drug repurposing platforms. Finally, as many CCC- and CCI-based predictions are context- and tissue-specific, generalizing findings across cancer types or patient populations remains a key hurdle. Overcoming these challenges will require tighter integration between computational modeling, experimental validation, medicinal chemistry, and clinical trial design.

Translational limitations further complicate clinical adoption. While several studies have proposed CCI-/CCC-based biomarkers, few have demonstrated robust predictive power or therapeutic benefit in prospective clinical trials. The integration of spatial and single-cell data into routine clinical workflows is also hindered by high costs, assay complexity, and infrastructure demands. Additionally, ethical and regulatory frameworks for large-scale patient-level data integration are still evolving. Issues of patient privacy, consent, and algorithmic transparency must be addressed to ensure responsible and equitable deployment of CCI-guided diagnostics and treatments.

In summary, realizing the full potential of CCI and CCC analyses in cancer research will require coordinated efforts to enhance methodological rigor, improve data integration and validation pipelines, expand drug development strategies, and create clinical frameworks that enable real-world translation.

### Towards clinical applications

These technologies are also able to examine patterns of CCI/CCC and the complexity of the tumor/stromal interface. For example, the tumor immune atlas combined single-cell and spatial transcriptomics data, identifying colocalization patterns of immune, stromal, and cancer cells in tumor sections, and correlating them with prognosis and immunotherapy response [98]. Integration of modern histological imaging with AI has also facilitated the evaluation of spatial immune cell density, heterogeneity, and clustering of immune cells. It also examines patterns of CCC and the complexity of the tumor/stromal interface. To achieve clinical adoption, assay standardization and multi-site studies need to be conducted. For example, the MITRE study assessed an mIF panel across six sites equipped with auto stainers and multispectral imaging systems, with analysis conducted at a single site, and concluded that high intersite and intrasite reproducibility was achievable, evidenced by high concordance in measurements of immune cell subsets, marker co-expression, and proximity [99].

In addition to the critical work on assay standardization and multi-site validation, biomedical researchers have an equally important role to play in driving CCI-based tools toward clinical adoption. First, it will be essential to develop and share robust, reproducible CCI inference pipelines, including open-source code, standardized input/output formats, and clear documentation so that independent groups can benchmark and extend these methods. Second, functional validation of predicted L-R interactions (for example, organoid co-culture systems, CRISPR perturbations, or microfluidic “tumor-on-a-chip” assays) will build the mechanistic evidence needed to convince translational teams of their therapeutic relevance. Third, integrating CCI metrics directly into preclinical drug-screening workflows is essential. For instance, using high-content imaging or spatial transcriptomics readouts will demonstrate how CCI features can predict compound efficacy or resistance.

Looking ahead, we predict that the integration of AI-driven analytical tools with extensive single-cell and spatial CCI/CCC data repositories, e.g., MOSAIC (Multi-Omic Spatial Atlas in Cancer) [100] will underpin the discovery and validation of CCI/CCC in drug development pipelines. Realizing this potential will require sustained collaboration across disciplines, including pathology, oncology, computational biology, and immunology. By combining robust assay platforms, reproducible computational pipelines, and functional validation strategies, the field can move toward embedding CCI/CCC insights directly into translational research and drug development workflows, maximizing clinical impact.

## Data Availability

No datasets were generated or analysed during the current study.

## Abbreviations

AI: Artificial intelligence
αSMA: Alpha smooth muscle actin
CAF: Cancer-associated fibroblast
CCC: Cell-cell colocalization
CCI: Cell-cell interaction
CD: Cluster of differentiation
CeDR: Cellular Drug Response Atlas
CHEA: ChIP-X Enrichment Analysis Database
CMAP: Connectivity Map
CRC: Colorectal cancer
CRISPR: Clustered Regularly Interspaced Short Palindromic Repeats
CROST: Comprehensive Repository Of Spatial Transcriptomics
CODEX: Co-detection by indexing
CTCL: Cutaneous T cell lymphoma
CTLA-4: Cytotoxic T-lymphocyte antigen 4
CTRPv2: Cancer Therapeutics Response Portal
CXCL9: C-X-C Motif Chemokine Ligand 9
CyTOF: Cytometry by Time of Flight
DEPOD: Dephosphorylation Database
DGIdb: Drug Gene Interaction Database
DNA: Deoxyribonucleic acid
ENCODE: Encyclopedia of DNA Elements
EVEX: Event Extraction from Literature with Multi-Level Gene Normalization
FOXP3: Forkhead Box P3
GCN: Graph Convolutional Neural Networks
GDSC: Genomics of Drug Sensitivity in Cancer
GEO: Gene Expression Omnibus
H&E: Hematoxylin and eosin
HCA: Human Cell Atlas
HER2: Human epidermal growth factor receptor 2
HNSCC: Head and neck squamous cell carcinoma
HPA: Human Protein Atlas
HTAN: Human Tumor Atlas Network
HTRIDB: Human Transcriptional Regulation Interaction Database
HuBMAP: Human BioMolecular Atlas Program
JASPAR: JASPAR Transcription Factor Binding Profile Database
KEGG: Kyoto Encyclopedia of Genes and Genomes
KEA: Kinase Enrichment Analysis Database
ICI: Immune checkpoint inhibitor
IF: Immunofluorescence
IHC: Immunohistochemistry
IMC: Imaging mass cytometry
IFN-I: Type I interferons
IUPHAR: International Union of Basic and Clinical Pharmacology
LDH: Lactate dehydrogenase
L-R: Ligand-receptor
LigRecDB: Ligand-Receptor Database
LIPSTIC: Labeling Immune Partnerships by SorTagging Intercellular Contacts
MIBI: Multiplexed ion beam imaging
mIF: Multiplex immunofluorescence
MISTy: Multi-Modal Imputation for Single-cell Transcriptomics
MOSAIC: Multi-Omic Spatial Atlas in Cancer
MOTIFMAP: Motif Map Database
mRNA: Messenger ribonucleic acid
MSigDB: Molecular Signatures Database
Ontogenet: Ontology-based Gene Expression and Network Analysis Tool
PD-1: Programmed death-1
PD-L1: Programmed death-ligand 1
PhosphoSite: PhosphoSite Database
PLA: Proximity Ligation Assay
PIC-seq: Sequence Physically Interacting Cells
RecTFDB: Receptor-Transcription Factor Database
RNA: Ribonucleic acid
RNA-seq: RNA sequencing
scRNA-seq: Single-Cell RNA sequencing
SCoPE-MS: Single Cell ProtEomics by Mass Spectrometry
SHP2: Src Homology 2 Domain-Containing Protein Tyrosine Phosphatase
SORC: Spatial omics resource of cancer
SOAR: Spatial transcriptOmics Analysis Resource
SpaOTsc: Spatially Optimal Transporting the Single Cells
SpiCi: Spatial Proteomics inferred Cell identification
SPIAT: Spatial Image Analysis of Tissues
ST: Spatial transcriptomics
STAT1: Signal transducer and activator of transcription 1
TF: Transcription factor
TFTGDB: Transcription Factor-Target Genes Database
TF_TG_TRRUSTv2: Transcription Factor-Target Gene Database
TGF-β: Transforming growth factor beta
TNFR: Tumor necrosis factor receptor
TIM-3: T-Cell Immunoglobulin and Mucin-domain containing-3
TTD: Therapeutic Target Database
TME: Tumor microenvironment
TNBC: Triple-negative breast cancer
TRANSFAC: Transcription Factor Database
uLIPSTIC: Universal version of Labeling Immune Partnerships by SorTagging Intercellular Contacts
VEGF: Vascular endothelial growth factor
VISTA: V-Domain Immunoglobulin Suppressor of T cell Activation
